# A simple and fast method for extraction and quantification of cryptophyte phycoerythrin

**DOI:** 10.1016/j.mex.2017.06.002

**Published:** 2017-06-28

**Authors:** Christina Thoisen, Benni Winding Hansen, Søren Laurentius Nielsen

**Affiliations:** Roskilde University, Department for Science and Environment, Universitetsvej 1, Postbox 260, Building 11.2, DK-4000 Roskilde, Denmark

**Keywords:** Labor cost, Microalgae, *Rhodomonas salina*, Pigment, Phycobiliprotein, Processing time

## Abstract

The microalgal pigment phycoerythrin (PE) is of commercial interest as natural colorant in food and cosmetics, as well as fluoroprobes for laboratory analysis. Several methods for extraction and quantification of PE are available but they comprise typically various extraction buffers, repetitive freeze-thaw cycles and liquid nitrogen, making extraction procedures more complicated. A simple method for extraction of PE from cryptophytes is described using standard laboratory materials and equipment. The cryptophyte cells on the filters were disrupted at −80 °C and added phosphate buffer for extraction at 4 °C followed by absorbance measurement. The cryptophyte *Rhodomonas salina* was used as a model organism.

• Simple method for extraction and quantification of phycoerythrin from cryptophytes.
• Minimal usage of equipment and chemicals, and low labor costs.
• Applicable for industrial and biological purposes.

## Graphical abstract

**Figure:**
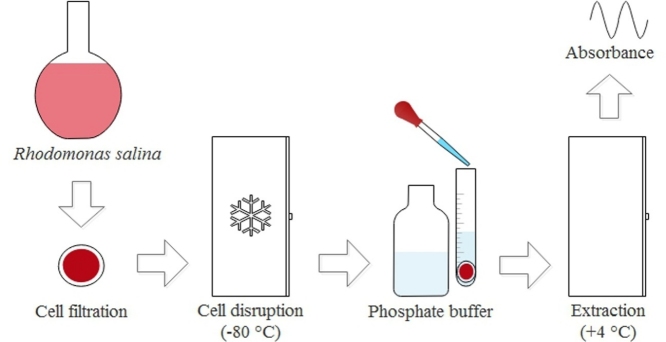

## Background

Phycoerythrin (PE) is a light harvesting pigment belonging to the phycobiliproteins, which also include phycocyanin, allophycocyanin and phycoerythrocyanin. Phycobiliproteins are found in red algae, cryptophytes and cyanobacteria [1], and are used as natural colorant in food and cosmetics. In particular, phycoerythrin is used as a fluoroprobe for clinical and biological analysis due to its high fluorescence [1].

The cell content of PE in microalgae depends on species and cultivation conditions. Generally, microalgae sustain cellular growth/metabolism during nitrogen limitation by degradation of phycobiliproteins [2], [3] which are nitrogen-rich. As an example, a study by Eriksen and Iversen [4] showed that nitrogen-sufficient cells of the cryptophyte *Rhodomonas* sp. were red and contained PE, while nitrogen-limited cells were green and without detectable amounts of PE. Also, the cell content of PE in *R. salina* was lower during nutrient limited cultivation compared to nutrient excess in a study by Vu et al. [5]. According to Kathiresan et al. [6], the content of PE in the red microalgae *Porphyridium purpureum* depends not only on nitrogen but on various specific macro nutrients. Light intensity and temperature can also affect the cell content of PE as demonstrated by Chaloub et al. [7] where PE in *Rhodomonas* sp. increased at low light intensity (15 μmol m^−2^ s^−1^, 12:12 light: dark cycle) combined with increased temperature (26 °C). Thus, quantification of the microalgal cell content of PE has relevant purposes such as being a proxy for the nutrient status during cultivation, and optimizing cultivation conditions to yield a higher cell content of PE.

The extraction efficiency of PE from microalgae depends on the rigidity of the cell wall, if present. The most suitable cell disruption method is therefore species dependent [8]. Cryptophytes do not possess a cell wall but a periplast of thin and fragile rectangular plates underneath the plasma membrane, which is very fragile (see references in Goldman and Dennett [9]) and easily disrupted. Numerous methods for extraction of PE are available but they are based on various species and comprise unnecessary chemicals, working steps and equipment for the extraction of PE from species without a cell wall [6], [10], [7]. The methods are often too comprehensive and time consuming for simple purposes such as comparing the cell content of PE whether it is between species/strains or between different cultivation conditions. For such comparisons it is beneficial with a simple and low labor cost method to obtain fast results.

This paper describes a simple and fast method for extraction and quantification of PE from the cryptophyte *R. salina* using only few materials and equipment easily available in standard laboratories at a low labor cost.

## Method details

### Materials

• Culture of *Rhodomonas salina*
• Whatman™ GF/C filter (0.2 μm)
• Pyrex glass vials
• Phosphate buffer (0.1 M, pH 6.7)
• Pasteur glass pipettes
• Syringe with 25 mm syringe filter (0.2 μm cellulose acetate membrane)
• Plastic cuvettes

### Equipment

• Filtration apparatus
• Refrigerator (+4 °C)
• Freezer (−80 °C)
• Spectrophotometer

## Pigment extraction and absorbance measurement

Filter the microalgae cells onto Whatman™ GF/C filters (0.2 μm) under a pressure of approximately 34 kPa. Fold the filters midway with the cells inside and transfer each filter to a Pyrex glass vial. Add 3 ml of the extraction solvent 0.1 M phosphate buffer (pH 6.7, 0.05 M K_2_HPO_4_, 0.05 M KH_2_PO_4_) and freeze at −80 °C for 24 h to disrupt the cells. Hereafter place the Pyrex glass vials with the filters in a refrigerator (4 °C) and extract for 24 h. Then transfer the extraction solvent with a Pasteur glass pipette (150 mm) to a 5 ml syringe with a 25 mm filter (0.2 μm, cellulose acetate membrane) and filter into a disposable plastic cuvette (1 cm path length). Measure the absorbance at 455, 564, 592 and 750 nm on a spectrophotometer using phosphate buffer as a blank. Scatter-correct the absorbance values by subtracting the absorbance at 750 nm. Avoid excess light exposure of the samples during the entire process from filtration to absorbance measurement by wrapping in, e.g., tin foil.

Calculate the content of phycoerythrin (PE) according to Beer and Eshel [11]:

PE (mg/ml) = [(*A*_564_ − *A*_592_) − (*A*_455_* − A*_592_)*0.2]*0.12

Where *A* refers to absorption at the indicated wave lengths.

## Additional information

To identify the easiest and fastest method for extraction and quantification of PE from *R. salina*, several treatments were compared (Table 1); freezing at −80 °C, lyophilization at −10 °C (Christ Alpha 1–2), and extraction in phosphate buffer each had a duration of 24 h. Sonication (Bransonic, Branson 1210, Struers KEBO Lab, model B1210E-MT) in an ice-bath with a frequency of 47 kHz ± 6% had a duration of 10 min. All samples were extracted for 24 h at 4 °C, and measured and calculated according to Beer and Eshel [11] as described in the previous section. All treatments used few materials and equipment available in standard laboratories. Replicates of all treatments were obtained at the same time from the same culture of *R. salina* cultivated at a low light intensity of 13 μmol m^−2^ s^−1^ and 17 °C.

**Table 1:** The treatments of filters with *R. salina* (1–8) and their processing time (days) used to identify the easiest and fastest method for extraction and quantification of PE.

Treatment	Day 0	Day 1	Day 2	Day 3
1	Extract	*Measure absorbance*		
2	Sonicate, extract	*Measure absorbance*		
3	Lyophilize	Extract	*Measure absorbance*	
4	Lyophilize	Sonicate, extract	*Measure absorbance*	
5	Freeze	Extract	*Measure absorbance*	
6	Freeze	Sonicate, extract	*Measure absorbance*	
7	Freeze	Lyophilize	Extract	*Measure absorbance*
8	Freeze	Lyophilize	Sonicate, extract	*Measure absorbance*

**Fig. 1:**
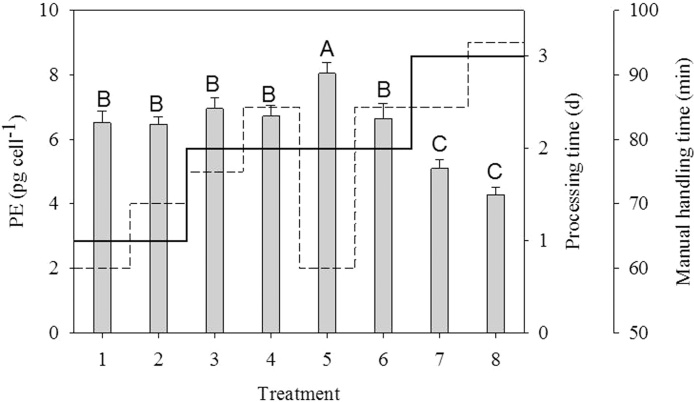
The cell content of PE (bars) in *Rhodomonas salina* from the treatments in Table 1. Treatment 5 resulted in the highest amount of PE, while treatment 7 and 8 resulted in the lowest amounts of PE (see text). Different letters indicate statistically significant differences between treatments. Bars are mean values ± S.D. (n = 3). The total processing time (d) for replicates of each treatment is indicated by the solid line. The estimated manual handling time (min) for replicates of each treatment is indicated by the dashed line. Manual handling time includes working steps such as filtrating cells onto filters and transferring filters to Pyrex glass vials.

There was a statistically significant difference in the yield of PE depending on the treatment (one-way ANOVA, F_7.16_ = 36.6, p < 0.001) (Fig. 1). Treatment 5 yielded a statistically significant higher amount of PE (8.04 ± 0.34 pg cell^−1^) compared to the other treatments (p ≤ 0.002, Holm-Sidak) with a processing time of 2 days. Therefore, treatment 5 is the recommended method described in the section above. However, the yield in treatment 5 was merely 19% higher compared to treatments 1 and 2 with a processing time of 1 day. Thus, methods with a processing time of 1 day are also applicable. A statistically significant lower cell content of PE (p ≤ 0.001, Holm-Sidak) was obtained with treatments 7 and 8.

Extraction of microalgal pigments often includes sonication and/or repetitive freeze-thaw cycles to disrupt the cell wall. Sonication, however, is unnecessary for cryptophytes since they do not have a cell wall. In fact, the presented results of PE for treatments exposed to sonication were lower compared to their counterpart without sonication (Treatment 1 versus 2, treatment 3 versus 4, etc.). Whether sonication has a direct negative effect on the yield of PE, or if this pattern is merely a coincidence, is unknown. Also, one freeze-thaw cycle using liquid nitrogen was sufficient for maximal PE extraction in *Pyrenomonas* (now *Rhodomonas*) *salina* [2], and a direct extraction of PE from *Rhodomonas* without any prior processing is possible as indicated by the results in Table 1 and Fig. 1.

Estimates of the manual handling time of triplicate samples of the treatments in Table 1 are shown in Fig. 1. This is defined as the time from which the cells are filtrated onto the filter to the PE results are obtained. The manual handling time includes working steps such as filtrating cells onto filters and transferring the filters to Pyrex glass vials, removing the lid from the Pyrex glass vials and adding phosphate buffer, etc. Based on the estimates, treatment 1 and 5 requires the lowest time of manual handling with 60 min. The total processing time of treatment 1 and 5 is 1 and 2 days, respectively (Fig. 1). In addition, the minimum extraction time (after one freeze-thaw cycle and 4 °C) of PE from *R. salina* was found to be 4 h by [12]. Thus, the processing times given above could likely be reduced by 20 h.

Based on the results for cell content of PE in the different treatments, the total processing time, and the manual handling time, we recommend the method for treatment 5. This method is a simple and fast method for obtaining results on the cell content of PE whether it is for comparing the content of PE in microalgal species, finding cultivation conditions resulting in a higher cell production of PE, or other comparative studies on the content of PE.
